# Invited commentary to ‘Acute pain management after thoracoscopic lung resection: a systematic review and explorative meta-analysis’

**DOI:** 10.1093/icvts/ivad015

**Published:** 2023-01-30

**Authors:** Lucio Cagini, Domenico Pourmolkara, Stefania Tamburrini, Mario Di Stasio

**Affiliations:** Thoracic Surgery Unit, Ospedale del Mare Napoli, University of Perugia, Naples, Italy; Thoracic Surgery Unit, Ospedale del Mare, Napoli, Italy; Radiology Unit, Ospedale del Mare, Napoli, Italy; Thoracic Surgery Unit, Ospedale del Mare, Napoli, Italy

**Keywords:** Video-assisted thoracic surgery, Pain management, Acute postoperative pain, Chronic postoperative pain

Pain after thoracoscopic surgery may increase the incidence of postoperative complications and impair the final results. Surprisingly, Cattoni *et al.* [1] reported that, after video-assisted thoracic surgery for the treatment of primary spontaneous pneumothorax, 3–63% of patients complain about the persistence of ipsilateral chest pain and/or paresthaesia, even several years after surgery [2].

In this study, Spaans *et al.* [3] performed a systematic review and meta-analysis to determine the mean pain scores of different analgesic techniques (thoracic epidural analgesia, continuous or single-shot unilateral regional analgesia and only systemic analgesia) after thoracoscopic anatomical lung resection.

In particular, they examined the clinical data of 5573 patients, calculating the mean pain score on a 0–10 scale at 24, 48 and 72 h after surgery; moreover, as a secondary outcome, they analysed postoperative nausea and vomiting, additional opioids, the use of rescue analgesia and length of hospital stay. The prediction of the pain level and its treatment is crucial because the incidence of chronic chest pain after video-assisted-thoracoscopic surgery must be considered [2].

From a cursory reading of this article, one may conclude that, with the same pain score, unilateral loco-regional techniques have a shorter length of stay and a lower incidence of postoperative nausea and vomiting than thoracic epidural analgesia; despite the outcomes have been calculated by a random-effect meta-analysis, and hence can guide us to credible conclusions, the authors point out that these conclusions cannot be drawn because the pooled results show an excessively high level of variability and heterogeneity between studies.

Adding the presence of confounding factors, such as study design (randomized controlled trial versus non-randomized controlled trial), different thoracoscopic approaches (single-port versus multi-port) and the existence of local practices of analgesic protocols, a meta-analysis and comparisons between different analgesics are unreliable.

Even though thoracic epidural analgesia in pain management in lung surgery has been used for decades, the recent PROSPECT guidelines support the loco-regional analgesic techniques discouraging the use of thoracic epidural analgesia due to its association with epidural haematomas and hypotension [4]. It has to be considered that, unlike this study, the PROSPECTS guidelines included patients with a majority not undergoing anatomical lung resection and did not perform a pooled meta-analysis.

In conclusion, the exciting issue examined in this study needs further investigation and more rigorous clinical evidence, with a well-designed randomized trial comparing loco-regional analgesia techniques to thoracic epidural analgesia to understand the best analgesic procedure that can benefit most patients undergoing thoracoscopic lung resections.
